# Association of Frailty With Transfusions, Hospitalizations, and Survival in Patients With Myelodysplastic Syndrome Initiating Hypomethylating Agents

**DOI:** 10.1002/jha2.70352

**Published:** 2026-07-29

**Authors:** Anahid Hamparsumian, Jennifer La, Mayuri Dharne, Gary Gilbert, Mary T. Brophy, Nhan V. Do, Dae Hyun Kim, Jane A. Driver, Nathanael R. Fillmore, Clark DuMontier

**Affiliations:** ^1^ Duke University School of Medicine Durham North Carolina USA; ^2^ Harvard Medical School Boston Massachusetts USA; ^3^ VA Boston Healthcare System Boston Massachusetts USA; ^4^ Chobanian and Avedisian School of Medicine Boston University Boston Massachusetts USA; ^5^ Marcus Institute for Aging Research Hebrew SeniorLife Boston Massachusetts USA; ^6^ Brigham and Women's Hospital Boston Massachusetts USA; ^7^ New England Geriatrics Research Education and Clinical Center VA Boston Healthcare System Boston Massachusetts USA; ^8^ Dana‐Farber Cancer Institute Boston Massachusetts USA

**Keywords:** aging, frailty, hypomethylating agents, myelodysplastic syndromes

## Abstract

**Introduction:**

Prognostic scoring systems in MDS lack functional status measures. We sought to evaluate the influence of frailty on overall survival and care utilization in a national cohort. We conducted a retrospective study in the US Veterans Affairs Healthcare System of patients with MDS initiated on hypomethylating agents (HMAs) from 2004 to 2023. Frailty was measured using the Veterans Affairs‐Frailty Index (VA‐FI).

**Methods:**

For primary analyses, we evaluated the association between severity of frailty and overall survival via Kaplan–Meier analysis, followed by Cox proportional hazard regression models. Multivariable Poisson regression was used to evaluate transfusion incidence and unplanned hospitalizations. We identified 2285 veterans with MDS who initiated HMA treatment. The median age was 73.2 years. In addition, 34.0%, 30.6%, and 35.4% of veterans were classified as non‐frail, mildly frail, and moderate‐to‐severely frail, respectively.

**Results:**

Moderate‐to‐severely frail veterans had a 49% higher hazard of death (aHR, 1.49; 95% CI, 1.34–1.67), as well as a higher incidence of transfusions (aIRR, 1.61; 95% CI, 1.45–1.80) and hospitalizations (aIRR, 1.58; 95% CI, 1.51–1.64).

**Conclusion:**

Increasing frailty was associated with higher rates of death, hospitalizations, and transfusions. Further research is needed to characterize how MDS‐related contributors to frailty and non‐oncologic aging‐related factors mediate the increased risk of inferior outcomes in this population.

**Trial Registration:**

The authors have confirmed clinical trial registration is not needed for this submission

## 1 Introduction

Management of myelodysplastic syndromes (MDS) is primarily guided by disease severity, with the Revised International Prognostic Scoring System (IPSS‐R) serving as the standard for assessment and prediction of leukemia‐free survival (LFS) and overall survival (OS) [1, 2, 3]. More recently, the IPSS‐molecular (IPSS‐M) has further incorporated disease genetics alongside laboratory and marrow‐based markers [4]. However, neither MDS risk system includes a formal measure of patient general health status—a missing risk factor that explains a large degree of variation in treatment outcomes, especially in older adults who make up the growing majority of patients with MDS [5].

Frailty is a summary measure of health status that reflects reduced physiologic reserve and higher risk of adverse outcomes when faced with stressors such as MDS and its treatment [5, 6, 7, 8]. Previous studies have shown that frail patients with MDS at various stages have higher healthcare utilization [9] and mortality [10, 11, 12, 13] compared to fit patients. Evidence is more limited on the influence of frailty for patients with disease advanced enough to require treatment, particularly with respect to outcomes such as blood transfusions and unplanned hospitalizations. Greater understanding is needed regarding how MDS‐related factors and aging‐related, non‐oncologic factors contribute to frailty to jointly elevate the risk of complications during treatment.

The US veteran population is, on average, more frail and multimorbid than the general public [14, 15]. The nationally integrated VA Healthcare System collects data on all veterans who initiate treatment for MDS, and the electronic Veterans Affairs‐Frailty Index (VA‐FI) provides a unique opportunity to assess frailty in these patients and its role in identifying those with poor treatment outcomes [15]. Accordingly, we evaluated the VA‐FI and its association with transfusion incidence, hospitalizations, and mortality in a large national cohort of veterans with MDS requiring disease‐modifying therapy with a hypomethylating agent (HMA). We hypothesized that increased levels of frailty are associated with increased mortality and health care utilization.

## 2 Methods

### 2.1 Study Design and Population

We conducted a retrospective cohort study of all veterans diagnosed with MDS identified in the national VA Cancer Registry from July 2004 to July 2023 who were initiated on HMAs: azacitidine, decitabine, and decitabine/cedazuridine. The national VA Cancer Registry comprises data from 132 Veterans Affairs Medical Centers on cancer diagnoses and treatment submitted by registry staff from each locality [16, 17]. Data from veterans who did not receive treatment within the VA Healthcare System or did not begin treatment within the study period were excluded. To increase selection for veterans who were continuously utilizing the VA Healthcare System prior to MDS treatment, we required at least one visit each year in the 3 years prior to their initial treatment date [18]. Moreover, we excluded patients with an AML diagnosis on or before MDS diagnosis. This study was approved by the VA Boston Health Care System Institutional Review Board. As this was a retrospective study using EHR data, the IRB granted a waiver of informed consent and a waiver of HIPAA authorization in accordance with 45 CFR 46.116(f) and 45 CFR 164.512(i), as all requirements for a waiver were met. All data were handled in compliance with institutional policies and federal regulations governing the protection of human subjects.

### 2.2 Frailty and Covariates

Our main exposure was patient frailty on the date of treatment initiation, assessed using the VA‐FI. The VA‐FI is an electronic measure of frailty consisting of 31 aging‐related deficits derived from EHR and claims data assessed within 3 years prior to treatment initiation [15]. Based on the well‐established deficit‐accumulation model of frailty [19], the VA‐FI includes deficits in five primary health domains, including morbidity (14 deficits), function (8 deficits), cognition (3 deficits), sensory (3 deficits), and other (3 deficits). A final score is computed as the proportion of all possible deficits that are present in a patient. We used previously published cutoffs to classify veterans as non‐frail (VA‐FI < 0.2), mildly frail (VA‐FI 0.2–0.3), or moderate‐to‐severely frail (VA‐FI ≥ 0.3). The electronic VA‐FI allows for retrospective assessment of frailty in VA patients while maintaining content, predictive, and construct validity, including against clinical frailty assessments performed by geriatricians [15, 20, 21, 22].

Covariates measured at initiation of treatment included sociodemographic characteristics—age, sex, race/ethnicity, and rurality—and laboratory variables known to be prognostic and accessible in VA's structured laboratory data: hemoglobin, platelet count, and white blood cell (WBC) count [23], taking the latest value within 90 days leading up to and 7 days after the date of treatment initiation. Missing lab values were replaced with median imputation. There was < 1% missingness in hemoglobin and platelet values and ∼2.6% missingness for WBC count.

### 2.3 Outcomes

The primary outcome was OS, measured using vital status information in the VA Corporate Data Warehose (CDW, which provides 98.3% sensitivity and 97.6% exact agreement against the US National Death Index) [24]. Patients were followed from their treatment initiation date until death or the end of the study period, August 2023, after which they were censored. Secondary outcomes were the incidence of red blood cell (RBC) transfusions and unplanned hospitalizations. Hospitalizations included any unplanned admissions within the VA Healthcare System with a hospital stay ≥ 1 day [25]. Secondary outcomes were measured until death, loss of follow‐up (defined as 90 days without a VA encounter), or end of study period, after which patients were censored.

### 2.4 Statistical Analysis

We computed descriptive statistics overall and by frailty category. For our primary analyses, we evaluated the association between severity of frailty and OS via Kaplan–Meier analysis, followed by univariable and multivariable Cox proportional hazard regression models, adjusting for age, sex, race/ethnicity, region, rurality, and baseline hemoglobin, platelet, and leukocyte counts. For our secondary analyses, we evaluated the association between frailty severity and transfusion incidence and unplanned hospitalizations using multivariable Poisson regression, adjusting for the same covariates as in our Cox model. Two‐sided *p*‐values < 0.05 were considered statistically significant. To account for secular trends in MDS treatment and supportive care over the two‐decade study period, we performed a sensitivity analysis of our multivariable models with additional adjustment for treatment era, defined a priori as 2004–2010 (early adoption of HMAs, [26]), 2011–2017 (HMA standardization), and 2018–2023 (contemporary treatment era). To further examine the relative prognostic contribution of comorbidity burden versus MDS disease among frail veterans, we conducted an exploratory analysis calculating the Myelodysplastic Syndrome‐Specific Comorbidity Index (MDS‐CI) [42] using VA‐FI codes for cardiac, hepatic, pulmonary, renal disease, and prior malignancy, and evaluated its association with our primary outcome of mortality in the main multivariable Cox model adjusting for clinical covariates. Analyses were conducted in R 4.1.2. This manuscript adheres to recommended reporting as per the Strengthening the Reporting of Observational Studies in Epidemiology (STROBE) guidelines [27].

## 3 Results

### 3.1 Baseline Characteristics of Study Population

**FIGURE 1:**
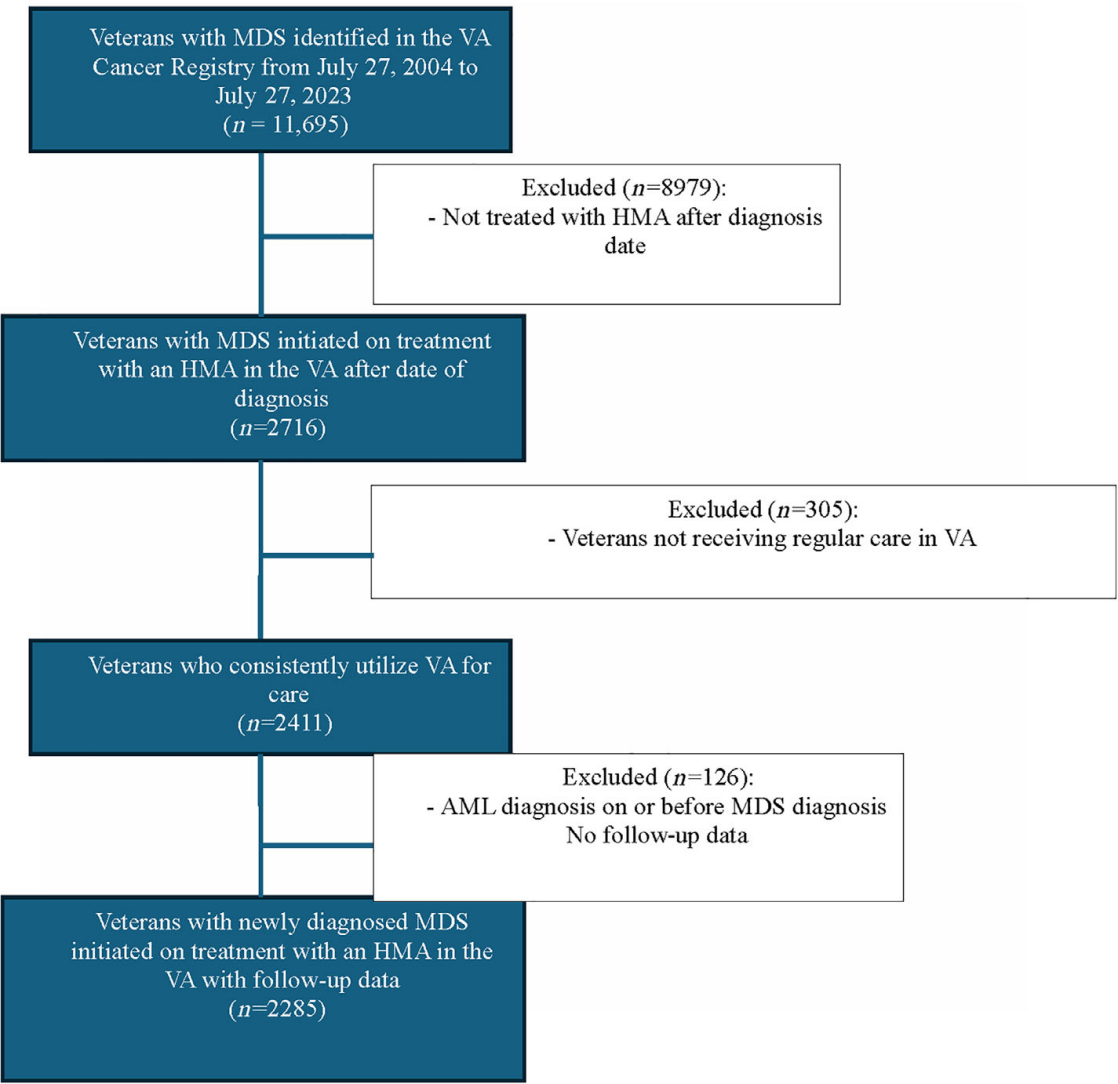
Flow diagram of selection of veterans with MDS newly treated in VA with disease‐modifying therapy.

We identified 2285 veterans initiated on disease‐modifying therapy for MDS with an HMA within the VA Healthcare System from July 2004 to July 2023 (Figure 1, Table 1). The median age was 73.2 years (IQR: 67.4–79.0), and the cohort was predominantly male (98.3%). The majority of patients were non‐Hispanic White (76.9%); 10.9% were African American, and 4.6% were Hispanic. In addition, 34.0%, 30.6%, and 35.4% of veterans were non‐frail, mildly frail, or moderate‐to‐severely frail, respectively (Table 1).

**TABLE 1:** Baseline characteristics of 2285 veterans with MDS initiated on disease‐modifying therapy with an HMA, according to frailty severity determined by VA‐FI.

	**Overall** **(*N* = 2285)**	**Non‐frail** **(*n* = 776)**	**Mild frailty** **(*n* = 700)**	**Moderate‐to‐severe frailty** **(*n* = 809)**
Age, median [IQR]	73.2 [67.4, 79.0]	71.9 [66.4, 77.4]	73.8 [68.4, 79.5]	74.0 [67.9, 79.8]
Sex				
Male	2247 (98.3%)	768 (99.0%)	689 (98.4%)	790 (97.7%)
Race/ethnicity, *n* (%)				
Non‐Hispanic White	1758 (76.9%)	607 (78.2%)	525 (75.0%)	626 (77.4%)
African American	249 (10.9%)	69 (8.9%)	81 (11.6%)	99 (12.2%)
Other or unknown	174 (7.6%)	72 (9.3%)	51 (7.3%)	51 (6.3%)
Hispanic	104 (4.6%)	28 (3.6%)	43 (6.1%)	33 (4.1%)
Region, *n* (%)				
North Atlantic	476 (20.8%)	143 (18.4%)	156 (22.3%)	177 (21.9%)
Midwest	595 (26.0%)	202 (26.0%)	174 (24.9%)	219 (27.1%)
Continental	369 (16.1%)	142 (18.3%)	110 (15.7%)	117 (14.5%)
Southeast	456 (20.0%)	162 (20.9%)	140 (20.0%)	154 (19.0%)
Pacific	389 (17.0%)	127 (16.4%)	120 (17.1%)	142 (17.6%)
Rurality, *n* (%)				
Urban	1492 (65.3%)	492 (63.4%)	458 (65.4%)	542 (67.0%)
Rural	734 (32.1%)	260 (33.5%)	223 (31.9%)	251 (31.0%)
N/A	32 (1.4%)	10 (1.3%)	12 (1.7%)	10 (1.2%)
Highly rural	27 (1.2%)	14 (1.8%)	7 (1.0%)	6 (0.7%)
Histology, *n* (%)				
MDS, NOS	1268 (55.5%)	422 (54.4%)	379 (54.1%)	467 (57.7%)
MDS/MPN, unclassifiable	78 (3.4%)	23 (3.0%)	24 (3.4%)	31 (3.8%)
MDS with 5q deletion	66 (2.9%)	16 (2.1%)	24 (3.4%)	26 (3.2%)
Therapy‐related MDS, NOS	18 (0.8%)	8 (1.0%)	6 (0.9%)	4 (0.5%)
MDS with RS and multilineage dysplasia	11 (0.5%)	2 (0.3%)	6 (0.9%)	3 (0.4%)
Chronic myelomonocytic leukemia, NOS	1 (0.0%)	0 (0.0%)	0 (0.0%)	1 (0.1%)
Neoplasm, malignant	1 (0.0%)	0 (0.0%)	0 (0.0%	1 (0.1%)
Labs, median [IQR]				
Median hemoglobin (Hgb, g/dL)	8.6 [7.7, 9.7]	8.80 [7.90, 9.90]	8.6 [7.8, 9.7]	8.4 [7.5, 9.6]
Median WBC (K/µL)	3.2 [2.1, 5.7]	3.02 [2.0, 4.9]	3.20 [2.1, 5.9]	3.4 [2.2, 6.1]
Median platelet count (K/µL)	68.0 [34.0, 133.0]	71.0 [36.0, 149.0]	62.0 [31.0, 128.3]	67.0 [33.0, 123.0]
Treatment, *n* (%)				
Azacitidine	1871 (81.9%)	646 (83.2%)	584 (83.4%)	641 (79.2%)
Decitabine	341 (14.9%)	108 (13.9%)	95 (13.6%)	138 (17.1%)
Cedazuridine/decitabine	73 (3.2%)	22 (2.8%)	21 (3.0%)	30 (3.7%)

Abbreviations: MDS, myelodysplastic syndromes; MPN, myeloproliferative neoplasm; NOS, not otherwise specified; RS, ringed sideroblasts; WBC, white blood cell count.

Median hemoglobin level at baseline was 8.8 g/dL (IQR: 7.9–9.90) in non‐frail patients compared to 8.4 g/dL (IQR: 7.5–9.6) in patients with moderate‐to‐severely frailty. Median WBC was 3.0 (IQR: 2.0–4.9) in the non‐frail group versus 3.4 (IQR: 2.2–6.1) in the group with moderate‐to‐severely frailty.

The most common first‐line HMA that patients were initiated on was single‐agent azacitidine for 1871 (81.9%) patients, whereas only 14.9% of patients received first‐line treatment with decitabine. Compared to non‐frail veterans, a lower percentage of moderate‐to‐severely frail veterans were started on azacitidine (83.2% vs. 79.2%), whereas a slightly higher percentage were started on decitabine (17.1% vs. 13.9%).

### 3.2 Deficits Contributing to Frailty

The prevalence of numerous health deficits contributing to frailty increased substantially across levels of frailty (Table 2). Compared to non‐frail veterans, moderate‐to‐severely frail veterans had higher rates of cardiovascular disease (CAD: 64.4% vs. 22.8%; heart failure: 41.8% vs. 4.9%; stroke: 29.0% vs. 5.7%), lung disease (60.1% vs. 21.8%), and chronic kidney disease (45.6% vs. 6.7%). Nearly half of moderate‐to‐severely frail veterans had depression (48.7%), and over a quarter had dementia (27.1%). There were also high rates of functional impairment: 40.2% of moderate‐to‐severely frail veterans required some prescription for durable medical equipment (e.g., a cane or walker), 44.1% had a gait abnormality, and 43.8% had muscle dysfunction (vs. 11.7%, 3.4%, and 3.1% in non‐frail patients, respectively). Prevalence of constitutional symptoms also varied. Fatigue was present in 54.3% of moderate‐to‐severely frail patients compared to 9.8% of non‐frail patients. Weight loss was identified in 21.1% of moderate‐to‐severely frail patients, compared to 4.4% of non‐frail patients.

**TABLE 2:** Prevalence of VA‐FI health deficits for 2285 veterans with MDS initiated on disease‐modifying therapy with an HMA, overall and according to frailty severity.

**Frailty deficit**	**Overall** **(*N* = 2285)**	**Non‐frail** **(*n* = 776)**	**Mild frailty** **(*n* = 700)**	**Moderate‐to‐severely frailty** **(*n* = 809)**
**Morbidity**				
Atrial fibrillation	465 (20.4)	51 (6.6)	138 (19.7)	276 (34.1)
Anemia	2184 (95.6)	698 (89.9)	684 (97.7)	802 (99.1)
Coronary artery disease	1013 (44.3)	177 (22.8)	315 (45.0)	521 (64.4)
Cancer^a^	1139 (49.8)	243 (31.3)	365 (52.1)	531 (65.6)
Cerebrovascular disease	398 (17.4)	44 (5.7)	119 (17.0)	235 (29.0)
Diabetes	936 (41.0)	181 (23.3)	271 (38.7)	484 (59.8)
Heart failure	492 (21.5)	38 (4.9)	116 (16.6)	338 (41.8)
Hypertension	1886 (82.5)	531 (68.4)	593 (84.7)	762 (94.2)
Incontinence	120 (5.3)	10 (1.3)	22 (3.1)	88 (10.9)
Kidney disease	570 (24.9)	52 (6.7)	149 (21.3)	369 (45.6)
Liver disease	357 (15.6)	51 (6.6)	95 (13.6)	211 (26.1)
Lung disease	927 (40.6)	169 (21.8)	272 (38.9)	486 (60.1)
Osteoporosis or pathologic fracture	117 (5.1)	14 (1.8)	34 (4.9)	69 (8.5)
Thyroid	346 (15.1)	70 (9.0)	103 (14.7)	173 (21.4)
**Function**				
Arthritis	1131 (49.5)	253 (32.6)	350 (50.0)	528 (65.3)
Durable medical equipment	587 (25.7)	91 (11.7)	171 (24.4)	325 (40.2)
Falls	204 (8.9)	21 (2.7)	43 (6.1)	140 (17.3)
Fatigue	697 (30.5)	76 (9.8)	182 (26.0)	439 (54.3)
Gait abnormality	483 (21.1)	26 (3.4)	100 (14.3)	357 (44.1)
Muscle dysfunction	472 (20.7)	24 (3.1)	94 (13.4)	354 (43.8)
Parkinson's disease	92 (4.0)	11 (1.4)	24 (3.4)	57 (7.0)
Peripheral vascular Disease	732 (32.0)	111 (14.3)	195 (27.9)	426 (52.7)
**Cognition and mood**				
Anxiety	418 (18.3)	55 (7.1)	115 (16.4)	248 (30.7)
Dementia	288 (12.6)	21 (2.7)	48 (6.9)	219 (27.1)
Depression	698 (30.5)	109 (14.0)	195 (27.9)	394 (48.7)
**Sensory function**				
Hearing impairment	858 (37.5)	179 (23.1)	279 (39.9)	400 (49.4)
Peripheral neuropathy	386 (16.9)	25 (3.2)	81 (11.6)	280 (34.6)
Vision impairment	585 (25.6)	118 (15.2)	169 (24.1)	298 (36.8)
**Constitutional**				
Chronic pain	502 (22.0)	64 (8.2)	133 (19.0)	305 (37.7)
Failure to thrive	39 (1.7)	1 (0.1)	6 (0.9)	32 (4.0)
Weight loss	288 (12.6)	34 (4.4)	83 (11.9)	171 (21.1)

^a^ These patients primarily had a diagnosis of prostate, colon, lung cancer, or MDS/AML, the latter being diagnosed in the years before initiation on HMA.

### 3.3 Frailty and Survival After Treatment Initiation

**FIGURE 2:**
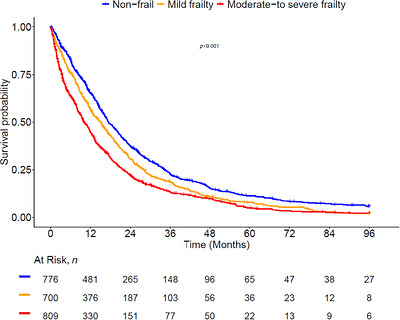
Kaplan–Meier analyses evaluating differences in overall survival by frailty severity (VA‐FI).

A total of 2005 patients (87.7%) died during the study period, with a median survival of 14.1 months (95% confidence interval [CI]: 13.2–14.9 months). Survival decreased with increasing frailty (log‐rank *p*‐value < 0.001, Figure 2): non‐frail veterans had the longest survival (median survival 17.4 months, 95% CI: 16.3–19.5 months), whereas moderate‐to‐severely frail veterans had the shortest (median survival 10.0 months; 95% CI: 9.3–11.6 months). Increasing frailty was associated with increasing mortality in univariable (Table S1) and multivariable Cox regression (Table 3), adjusting for covariates including age, sex, race/ethnicity, region, rurality, and baseline hemoglobin, platelet, and leukocyte counts. Compared to non‐frail veterans, moderate‐to‐severely frail veterans had 1.49 times higher mortality (hazard ratio [HR], 1.49; 95% CI, 1.34–1.67). Complete case analysis, excluding those who had missing laboratory values, showed similar results (Table S2), as did the sensitivity analysis further adjusting for treatment era (Table S3). The exploratory analysis of the MDS‐CI showed increasing hazard of mortality with increasing comorbidity burden (Table S4).

**TABLE 3:** Multivariable Poisson and Cox proportional hazards models estimating the association of frailty severity (VA‐FI) with incidence of RBC transfusion, unplanned hospitalization, and mortality.

		RBC transfusion incidence		Hospitalizations		Mortality	
Frailty severity	*N* (%)	IRR	95% CI	IRR	95% CI	HR	95% CI
Non‐frail (VA‐FI < 0.2)	776 (34.0%)	Ref	Ref	Ref	Ref	Ref	Ref
Mild frailty (VA‐FI 0.2–0.3)	700 (30.6%)	1.14	1.03–1.27	1.03	0.98–1.07	1.12	1.0–1.24
Moderate‐to‐severely frailty (VA‐FI ≥ 0.3)	809 (35.4%)	1.61	1.45–1.80	1.58	1.51–1.64	1.49	1.34–1.67

*Note*: Models adjusted for age, sex, race/ethnicity, region, rurality, and baseline hemoglobin, platelet, and leukocyte counts.

Abbreviations: CI, confidence interval; HR, hazard ratio; IRR, incidence rate ratio.

### 3.4 Frailty and Blood Transfusions, Unplanned Hospitalizations

Over 3330 total person‐years of follow‐up after treatment initiation, patients experienced a total of 2444 blood transfusions and 16,742 unplanned hospitalizations after treatment initiation. Moderate‐to‐severely frail veterans had an average of 2.36 RBC transfusions per 5 person‐years, compared to 1.34 RBC transfusions in non‐frail veterans (adjusted incidence rate ratio [aIRR], 1.61; 95% CI, 1.45–1.8; Table 3). Moderate‐to‐severely frail veterans had an average of 3.89 hospitalizations per 5 person‐years, compared to 2.35 hospitalizations per 5 person‐years in non‐frail veterans (aIRR, 1.57; 95% CI, 1.51–1.63; Table 3). Complete case analysis, excluding those who had missing laboratory values, showed similar results (Table S2), as did the sensitivity analysis further adjusting for treatment era (Table S3).

## 4 Discussion

In US veterans with MDS advanced enough to initiate disease‐modifying treatment with an HMA, we found that overall median survival was 14.1 months and that increasing severity of frailty was associated with higher mortality, independently of sociodemographic covariates and laboratory markers of MDS severity. Moreover, increasing frailty was associated with a significantly higher rate of blood transfusions and unplanned hospitalizations. Frailty may be a significant factor explaining the lower survival in real‐world analyses of patients with MDS treated with azacitidine, as compared to the 24.4‐months median shown in the AZA‐001 trial [26, 28, 29]. These findings highlight the need to assess frailty—both MDS‐related deficits and non‐oncologic, aging‐related deficits—alongside disease risk in patients with MDS initiating treatment.

Two large European real‐world retrospective studies described a similar OS of 13.4–13.5 months for patients on azacitidine for higher‐risk MDS. Age and ECOG performance status ≥ 2 were found to be important prognostic factors, respectively. To date, our analysis of 2285 patients is the largest study investigating frailty in a population with MDS initiating an HMA. Prior studies have proven the predictive value of summary measures of frailty, shorter frailty instruments, or related measures of aging vulnerability in patients diagnosed with MDS at various stages in their disease course [10, 12, 30]. In a prospective cohort study of 445 patients with MDS enrolled a median of 6.1 months after diagnosis, Buckstein et al. measured frailty using the Rockwood Clinical Frailty Scale (CFS) and found it to be associated with survival independently of IPSS‐R [10]. Sakatoku et al. reinforced the association between CFS and survival in their retrospective analysis of 118 patients diagnosed with MDS [11]. Prior work has developed MDS‐specific frailty indices, the MDS‐FI and the shorter FS‐15, that incorporate both laboratory measures reflecting disease risk (e.g., leukocyte count and reticulocyte count) and geriatric assessment measures such as activities of daily living and 4‐m gait speed [12, 13]. In a regression‐based risk score derived from data in 440 patients diagnosed with MDS, the inclusion of either the MDS‐FI or FS‐15 added independent predictive value to survival beyond the IPSS. Although we cannot extract from VA‐structured data the cytogenetics and bone marrow reports necessary for calculation of IPSS, we adjusted our analyses for hemoglobin, WBC, and platelet levels to control for severity of disease. Moreover, the centralized VA database allowed us to identify all patients with MDS initiating disease‐modifying therapy, as a means of defining a higher‐risk group of patients for our cohort [10, 11, 12]. Specifically defining our cohort as patients newly initiated on an HMA allowed for observation of the impact of frailty at a uniform, clinically relevant decision point in the MDS disease course, while also controlling for several MDS disease‐related and sociodemographic pretreatment confounders, including age [31]. In this way, our findings provide greater understanding of the impact of frailty in patients receiving treatment with an HMA, as only about 15%–30% of patients in the above studies were reported to be on HMA therapy [10, 11, 12]. Although a comparison of frail treated versus untreated patients would be of clinical interest, this analysis would be subject to biases such as immortal time and confounding by indication that are difficult to overcome in a retrospective observational study [31].

Though the VA‐FI has not been directly compared with the aforementioned MDS‐specific measures, there are advantages of the electronic VA‐FI in measuring frailty in MDS. The VA‐FI is readily derived from existing data in the electronic medical record and has been validated against multiple clinical measures of frailty, which carry predictive value in other prospective cohorts of patients with cancer but require additional time and resources for implementation [20]. A decision‐support application has been developed to prospectively measure VA‐FI in oncology clinics, facilitating the translation of retrospective findings, such as those in this study, to immediately inform patient care [32].

Moreover, the VA‐FI aggregates information for 3 years preceding the initiation of treatment in our study, as opposed to a static point in time. This 3‐year assessment period provides information on the non‐oncologic factors that contribute to frailty before MDS is diagnosed or treated. Compared to non‐frail veterans, veterans with the highest levels of frailty had a 3–10 times higher prevalence of comorbidities (across multiple organ systems), mood disorders and dementia, and functional deficits. These non‐oncologic health deficits could increase risk of complications by themselves or through interaction with MDS and its treatment.

For example, heart failure, chronic lung disease, and liver disease may increase the risk of hospitalization and death owing to less physiologic compensation for anemia or bleeding [33, 34, 35]. Interestingly, a recent retrospective analysis of the MDS‐CAN cohort demonstrated that frail patients (classified by the FS‐15) had a higher rate of death due to infections [36]. Frailty from both MDS and other comorbidities may explain the increased risk of infection described in older adults, in particular when compared to people of the same age without MDS, and the increased risk of death from infection in patients > 70 years old [37]. Madry et al. studied survival and infection risk based on data from 298 patients receiving treatment with azacitidine for MDS [38], acute myeloid leukemia (AML), or chronic myelomonocytic leukemia (CMML), and found both ECOG ≥ 2 and RBC transfusion dependency as predictors of infection. Preexisting malnutrition or failure to thrive may compound the gastrointestinal adverse effects and anorexia that can arise from HMA treatments [39, 40]. Mood and cognitive disorders may limit the ability to adhere to treatment schedules and follow‐up appointments [41]. Our exploratory analysis showing the association of increasing MDS‐CI [42], calculated using the corresponding comorbidity codes measured in the VA‐FI, adds further evidence to the prognostic importance of extra‐hematological comorbidities in our population. Importantly, frailty—while influenced by comorbidity—is a distinct construct, encompassing a broader state of vulnerability that also reflects impairment in other aging‐related domains such as functional, cognitive, sensory, and constitutional deficits [42]. Its prognostic importance rests not only on the influence of these individual health deficits, but also on their cumulative impact on physiologic reserve and the ability to compensate for the stressors of MDS and treatment.

Future research should characterize the specific types of adverse events driving hospitalizations and mortality in frail veterans and the role that these non‐oncologic health deficits play in real‐world care trajectories not captured among the patients treated in clinical trials. Reinforcing this need is our novel finding linking frailty with higher rates of blood transfusions after initiation of treatment. Certainly, as a patient's MDS progresses, they may become more frail from anemia, though in our study the median hemoglobin level was similar in each of the frailty subgroups. Moderate‐to‐severely frail veterans had higher rates of fatigue and muscle dysfunction compared to non‐frail veterans, signs and/or symptoms that can trigger orders for transfusions [14, 15, 43]. Moreover, moderate‐to‐severely frail veterans had higher rates of kidney disease, nutritional deficiency (weight loss), thyroid disease, and liver disease—all potential causes of anemia that could interact with marrow failure in MDS to drive lower hemoglobin levels and thus higher transfusion rates. One or more of the many comorbidities highly prevalent in frail patients could also provide rationale for keeping a higher transfusion goal or contraindicate use of other supportive therapies, such as erythrocyte‐stimulating agents (ESAs), for example, a history of CAD versus CVA. Finally, frailty at the time of treatment initiation could reflect prolonged progression of MDS, especially if the anemia, fatigue, and other frailty deficits are largely secondary to MDS versus alternative causes. Future work should investigate the clinical and biological mechanisms by which frailty is associated with increased transfusion requirements, delineating MDS‐ and non‐oncologic causes.

There are limitations to our study. As already mentioned, we were unable to measure potential confounding factors such as absolute neutrophil count, bone marrow blast percentage, and cytogenetics, which are used to generate the IPSS‐R but are not readily available in VA‐structured laboratory data. Future analyses could incorporate these variables using manual extraction from chart review or extraction via natural language processing/large language models. Since HMAs are first line for higher‐risk MDS, restricting our analyses to patients initiating treatment on HMAs improves homogeneity of higher‐risk MDS in our population without the need to account for reclassification of risk across different systems (e.g., IPSS‐R and IPSS‐M) [4, 44].

Moreover, we were unable to evaluate other prognostic factors such as time from MDS diagnosis to treatment initiation, duration of treatment, subsequent disease‐specific markers of treatment response, and subsequent treatment changes, limiting our evaluation of the degree to which these factors influenced the relationship between frailty and our outcomes of interest. Future studies should evaluate whether MDS treatment is delayed in frail patients due to tolerability concerns. Finally, external care utilization and competing risks may have introduced bias in our outcomes of RBC transfusion incidence and hospitalizations, although we do not have evidence to suggest that external care utilization varied by frailty severity. Cause‐specific mortality (MDS‐related vs. non‐oncologic) would be informative; however, reliable attribution of cause of death in MDS patients is not available in our administrative data and is known to be misclassified without adjudication [45]. Therefore, our analyses focus on all‐cause mortality, and future work with adjudicated cause‐of‐death data will be needed to evaluate competing causes of death.

## 5 Conclusion

Our findings link frailty with mortality, hospitalizations, and transfusions in a complex US population initiating treatment with an HMA for MDS. That the relationship between frailty and outcomes is independent of markers of MDS risk underscores the importance of integrating holistic measures of patient health status alongside disease‐specific measures such as the IPSS—especially for real‐world patients who are underrepresented in clinical trials [46, 47]. Further research is needed to characterize how MDS‐related and non‐oncologic, aging‐related factors mediate poorer outcomes in frail patients. Incorporation of the electronic VA‐FI in prospective studies of US veterans with MDS could reveal treatment modifications that effectively control disease without overwhelming physiologic reserve, while also guiding supportive interventions that aim to optimize aging‐related vulnerabilities regardless of the treatment chosen.

## Author Contributions

A.H., J.L., N.R.F, and C.D. conceptualized and designed the study. A.H., J.L., N.R.F., and C.D. performed data curation and quality control. J.L. performed data analysis. All authors contributed to the interpretation of results and manuscript writing.

## Conflicts of Interest

All authors declare no conflicts of interest.

## Supporting information




**Supporting file**: jha270352‐sup‐0001‐SuppMat.pdf

## Data Availability

The derived data generated in this research may be shared on reasonable request to the corresponding author as permitted by VA policy.
