# Supplementary material for: Association of Frailty With Transfusions, Hospitalizations, and Survival in Patients With Myelodysplastic Syndrome Initiating Hypomethylating Agents
Source: EJHaem. 2026 Jul 29;7(4):e70352. doi: 10.1002/jha2.70352 (PMC13418855; doi:10.1002/jha2.70352)
Supplement: Supplementary file 1 — Supporting file: jha270352‐sup‐0001‐SuppMat.pdf [file JHA2-7-e70352-s001.pdf]

Supplemental Table 1:

Univariable unadjusted analysis of RBC transfusion incidence, hospitalizations, and mortality by frailty severity level.

**Frailty Severity**	**N** (%)	**RBC Transfusion Incidence**	**Hospitalizations**	**Mortality**
		IRR (95% CI)	IRR (95% CI)	HR (95% CI)
**Non-frail** (VA-FI < 0.2)	776 (34.0%)	ref	ref	ref
**Mild frailty** (VA-FI 0.2-0.3)	700 (30.6%)	1.30 (1.18-1.44)	1.11 (1.07-1.16)	1.23 (1.10-1.37)
**Moderate-Severe frailty** (VA-FI ≥ 0.3)	809 (35.4%)	1.76 (1.59-1.95)	1.66 (1.60-1.72)	1.57 (1.41-1.74)
IRR = incidence rate ratio; HR = hazard ratio; CI = Confidence Interval				

Supplemental Table 2:

Multivariable analysis of RBC transfusion incidence, hospitalizations, and mortality by frailty severity level, conducted using complete case analysis (excluding patients with missing covariates).

**Frailty Severity**	**RBC Transfusion Incidence**		**Hospitalizations**		**Mortality**	
	IRR	95% CI	IRR	95% CI	HR	95% CI
**Non-frail** (VA-FI < 0.2)	ref	ref	ref	ref	ref	ref
**Mild frailty** (VA-FI 0.2-0.3)	1.14	1.03-1.27	1.03	0.98-1.07	1.10	0.98-1.24
**Moderate-Severe frailty** (VA-FI ≥ 0.3)	1.61	1.45-1.80	1.58	1.51-1.64	1.49	1.34-1.67
Models adjusted for age, sex, race/ethnicity, region, rurality, and baseline hemoglobin, platelet, and leukocyte counts.<br><br>IRR = incidence rate ratio; HR = hazard ratio; CI = Confidence Interval						

Supplemental Table 3: Multivariable Poisson and Cox proportional hazards models estimating the association of frailty severity (VA-FI) with unplanned hospitalization and mortality, further adjusted for treatment era.

**Frailty Severity**	**N (%)**	**Hospitalizations**		**Mortality**	
		IRR	95% CI	HR	95% CI
**Non-frail** (VA-FI < 0.2)	776 (34.0%)	ref	ref	ref	ref
**Mild frailty** (VA-FI 0.2-0.3)	700 (30.6%)	1.01	0.97-1.05	1.13	1.01-1.23
**Moderate-Severe frailty** (VA-FI ≥ 0.3)	809 (35.4%)	1.47	1.41-1.53	1.52	1.36-1.69
Models adjusted for age, sex, race/ethnicity, region, rurality, and baseline hemoglobin, platelet, leukocyte counts, and treatment era (2004-2010; 2011-2017; 2018-2023). Models of RBC transfusion incidence adjusted for treatment era were not performed because annual changes in transfusion procedural codes could bias such adjustment.<br><br>IRR = incidence rate ratio; HR = hazard ratio; CI = Confidence Interval					

Supplemental Table 4: Multivariable Cox proportional hazards models estimating the association between comorbidity severity (myelodysplastic syndrome-specific comorbidity index, MDS-CI) and mortality.

**MDS-CI**	**Mortality**	
	**HR**	**95% CI**
Low Risk (0)	ref	ref
Intermediate risk (1-2)	1.31	1.12 – 1.54
High Risk (≥3)	1.60	1.37-1.86
Models adjusted for age, sex, race/ethnicity, region, rurality, and baseline hemoglobin, platelet, and leukocyte counts.<br><br>HR = hazard ratio; CI = Confidence Interval		
